# A Fetus with Iniencephaly Delivered at the Third Trimester

**DOI:** 10.1155/2015/520715

**Published:** 2015-08-06

**Authors:** Esra Cinar Tanriverdi, Ilhan Bahri Delibas, Zeynep Kamalak, Berrin Goktug Kadioglu, Rukiye Ada Bender

**Affiliations:** ^1^Department of Gynecology and Obstetrics, Nenehatun Maternity Hospital, 25000 Erzurum, Turkey; ^2^Department of Obstetrics and Gynaecology, Faculty of Medicine, Gaziosmanpasa University, 60000 Tokat, Turkey; ^3^Buhara Hospital, 25000 Erzurum, Turkey

## Abstract

Iniencephaly is an uncommon neural tube defect, having retroflexion of the head without a neck and severe distortion of the spine. Iniencephaly is classified into two groups, iniencephaly apertus (with encephalocele) and iniencephaly clausus (without encephalocele). Incidence ranges from 0.1 to 10 in 10.000 pregnancies and it is seen more frequently in girls. Most of the fetuses with this defect die before birth or soon after birth, while those with the milder forms may live through childhood. Recurrence risk is around 1–5%. Family should be offered termination to reduce maternal risks and counseled for folic acid supplementation before the next planned pregnancy. Here we present a rare case of iniencephaly clausus which was diagnosed at 18th week of gestation by ultrasonography and delivered in the third trimester of pregnancy due to rejection of termination.

## 1. Introduction

Iniencephaly is a severe form of neural tube defect (NTD) with poor prognosis, characterized by shortening of spinal column, rotation, and retroflexion of the head and absence of the neck due to defective closure of the vertebral arches and bodies [1, 2]. While the exact etiopathogenesis is not known, genetic and environmental factors have been implicated. Environmental factors such as poor socioeconomic status, low parity, folic acid deficiency, obesity, and drugs including sulfonamide, tetracycline, antihistamine, and antitumor agents are shown to increase the risk. Trisomy 18, trisomy 13, and monosomy X have been associated with this disorder [1–3]. Iniencephaly is usually detected at the late first trimester by ultrasound due to overt postural abnormalities. The purpose of this case report was to remind the reader about this rare type of NTD and to show the feasibility of ultrasonography in the prenatal diagnosis.

## 2. Case Report

**Figure 1:**
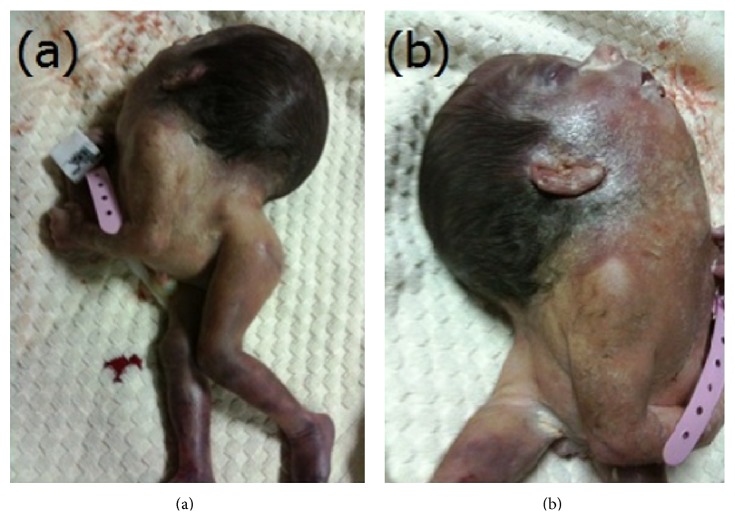
(a) Gross photograph of iniencephaly showing grotesque hyperextension of the head, short spine, and lack of neck (b) typical “star-gazing” appearance. Also note the low-set ears.

**Figure 2:**
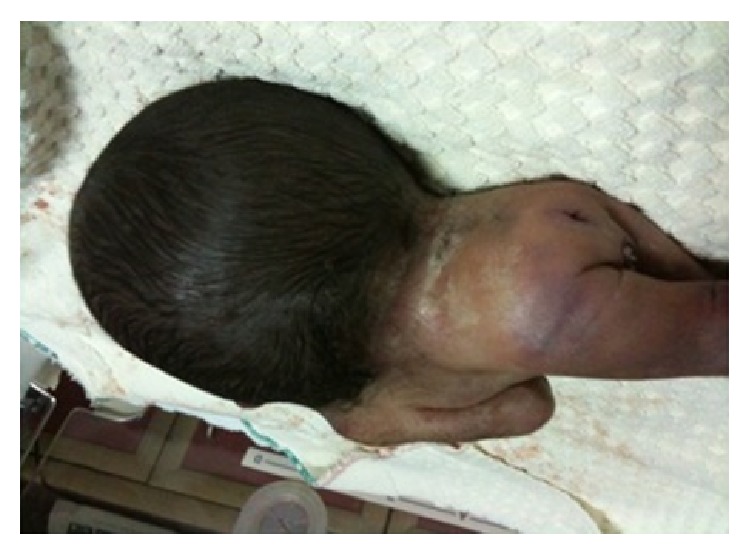
Lumbar spina bifida.

A 22-year-old gravida 2, para 1, living 1 woman presented to obstetrics polyclinic for her first obstetric visit. She had a history of nonconsanguineous marriage. She had no history of iron and folic acid supplementation. Her routine blood and biochemical tests were within normal limits. Abdominal examination revealed a uterus of 16-week size. Serum alpha-fetoprotein was significantly raised. Ultrasound revealed an 18-week, single-live fetus with severely hyperextended head and strikingly short vertebral column with no apparent fetal neck. The family was counseled and offered termination of the pregnancy. After rejecting termination, they were offered an amniocentesis, which they also rejected. The woman did not continue her routine visits. At 32 weeks of gestation, she was admitted with rupture of membranes and contractions. Ultrasonography revealed a live fetus with hydrocephaly and iniencephaly. Since biparietal diameter of the fetus was above 100 mm and the mother had a previous C-section, she underwent cesarean delivery. A 1350 gr female fetus with fixed retroflexion of the head, without a neck, and with low-set ears (Figure 1) and lumbar meningocele (Figure 2) was dead at 10 minutes after birth. Since the family did not accept autopsy, further evaluation for the presence of additional anomalies was impossible.

## 3. Discussion

Iniencephaly is an extremely rare neural tube closure defect. Its incidence ranges from 0.1 to 10 in 10.000 pregnancies. It is nearly 4 times more common in females and has a recurrence risk of 1–5% in next pregnancies [1]. The Greek word “inion” means back of the neck. Here inion (external occipital protuberance) joins with the back, leading to retroflexion of the head and absence of the neck. Persistence of the embryonic cervical lordosis at the third week, leading to failure of closure of the neural tube, and abnormal development of the rostral portion of the notochord and somites of the cervicooccipital region are the most widely accepted theories for its embryological development [1].

The criteria for the diagnosis of iniencephaly are (1) imperfect formation of the base of the skull, particularly at the level of the foramen magnum, (2) rachischisis, and (3) exaggerated lordosis of the spine [1]. The spine is short and grossly abnormal, with kyphoscoliosis [2]. It can be classified into two groups, based on the presence or absence of encephalocele, iniencephaly apertus and iniencephaly clausus. Prognosis is poor and majority of the newborns die within hours after birth. Prenatal diagnosis of iniencephaly by ultrasonography is relatively easy due to severe anomalies of axial anatomy and vertebrae and typical fetal position. Timely prenatal diagnosis and termination offer are particularly important to reduce maternal risks [1–3].

While the exact etiology of iniencephaly is not clear, genetic and environmental factors have been implicated. It was reported to be associated with trisomy 13, trisomy 18, monosomy X, partial monosomy 6p, and partial trisomy 11q [4]. Low socioeconomic status, low parity, folic acid deficiency, obesity, some drugs (sulfonamides, tetracyclines, antitumor agents, and antihistamines), and congenital syphilis have been shown to increase the risk [1, 5, 6]. In our case low socioeconomic status and lack of folic acid supplementation were present, which may be the cause. There was no history of drug use and treponemal tests were negative.

The two diagnostic clues on ultrasonography that readers can learn from the case are extreme dorsal flexion of the head and an abnormally short and deformed spine. Occipital bone defect leading to an enlarged foramen magnum, irregular fusion of malformed vertebrae, and incomplete closure of vertebral arches and bodies leading to retroflexion of cervical spine and upward turned face with chin continuous with chest because of the absence of neck (star-gazing appearance) are classical sonographic features for iniencephaly [7]. If ultrasound is insufficient in diagnosis, MRI or CT can be utilized. Magnetic resonance imaging is a better diagnostic tool to evaluate the anomalies in detail, especially the ones in the brainstem and the spine, and should be considered particularly in women whose pregnancy was not interrupted [8].

Two groups of anomalies lead to congenital retroflexion of spine, which are anencephaly and iniencephaly. Iniencephaly apertus can be differentiated from anencephaly with retroflexion of cervical spine. Cervical vertebrae are abnormal and retroflexed in iniencephaly while they are almost normal in anencephaly. Anencephaly shows an at least partial absence of neurocranium and no skin coverage of the retroflexed head while the retroflexed head is completely covered with skin in iniencephaly. In this case the retroflexed head was completely covered with skin without encephalocele.

Also, differential diagnosis of iniencephaly clausus should be made from Klippel-Feil syndrome (KFS) and cervical meningomyelocele [4]. Differentiation between iniencephaly clausus and KFS is important and appears to be difficult. A failure of segmentation of the cervical vertebrae during early fetal development leads to KFS. Despite the fact that abnormal and fused cervical vertebrae may be seen in both of these anomalies, in KFS, retroflexion of head and gross and devastating abnormalities of the spine are absent. In addition, KFS is not fatal and surgically correctable.

Anencephaly, microcephaly, hydrocephaly, holoprosencephaly, posterior fossa defects, and spinal defects may be seen together with iniencephaly. The most common accompanying anomalies are spina bifida (74%), diaphragmatic hernia (37%), adrenal hypoplasia (37%), club foot (32%), hypoplastic lung, single umbilical artery, omphalocele (26%), cardiovascular anomalies, genitourinary malformations, cyclopia, cleft lip and palate, and imperforated anus [9, 10].

Ultrasound is usually sufficient for diagnosis with a minimum cost and easy application. Careful ultrasonographic examination is usually effective in the diagnosis of iniencephaly during the first trimester, at the time of nuchal translucency measurement. Prenatal diagnosis has been reported to be possible as early as almost 9th gestational week by transvaginal USG [11].

Postural anomalies may cause obstructed labor and maternal trauma can occur due to hydrocephaly and extreme retroflexion of the head; therefore early diagnosis and termination of pregnancy should be offered to the parents before viability to reduce maternal risks [12–14]. Since iniencephaly has a recurrence rate of 1–5%, the women should be counseled for folic acid supplementation before future pregnancies.
